# Case Report: Transforming small cell lung cancer: two cases report and literature review

**DOI:** 10.3389/fonc.2025.1573624

**Published:** 2025-04-03

**Authors:** Jinlong Liu, Jing Ai, Lize Zhao, Yimeng Qian, Qingxin Zhao, Chunling Ma, Yu Zhao, Jing Zhao

**Affiliations:** ^1^ Department of Oncology, Hebei General Hospital, Shijiazhuang, China; ^2^ Graduate School, Hebei North University, Zhangjiakou, China; ^3^ Graduate School, North China University of Science and Technology, Tangshan, China; ^4^ Department of Vascular Surgery, Hebei General Hospital, Shijiazhuang, China

**Keywords:** transformed small cell lung cancer, tumor markers, re-biopsy, pathological transformation, drug resistance

## Abstract

Transformation of non-small cell lung cancer (NSCLC) to small cell lung cancer (SCLC) is a relatively rare event with an incidence of about 3%-14%. Due to the poor treatment outcomes and prognosis associated with this transformation, understanding its underlying mechanisms is crucial for improving the diagnosis, management, and prognosis of transformed small cell lung cancer (T-SCLC). In this paper, we present two cases of T-SCLC and review the molecular mechanisms, clinical features, and treatment strategies post-transformation. We emphasize the importance of considering pathological transformation in cases of resistance to NSCLC treatment, monitoring changes in tumor markers, and conducting a re-biopsy. Finally, we propose effective treatment measures for managing T-SCLC.

## Introduction

According to the Global Tumor Epidemiology Statistics (GLOBOCAN), lung cancer ranks first among malignant tumors in China in terms of both new cases and deaths (1). It is classified into two major subtypes based on histology: NSCLC and SCLC. SCLC is a neuroendocrine tumor characterized by high aggressiveness, malignancy, and poor prognosis. During the progression of NSCLC, its histological type can transform into SCLC, a phenomenon known as T-SCLC. Although SCLC and T-SCLC share similar pathological morphology, molecular features, clinical manifestations, and drug sensitivity, their pathogenesis and tumor microenvironment differ significantly. Effective treatments for T-SCLC remain limited, and its prognosis is poor. In this paper, we present two cases of T-SCLC and provide a comprehensive summary of the clinical features, mechanisms, predictive factors, and treatment strategies associated with SCLC transformation. Our findings emphasize the importance of re-biopsy in managing drug resistance and the necessity of dynamic monitoring of tumor marker changes. Furthermore, we propose an effective treatment approach for T-SCLC, offering new perspectives for clinical diagnosis and management.

## Case presentation

### Case 1

**Figure 1:**
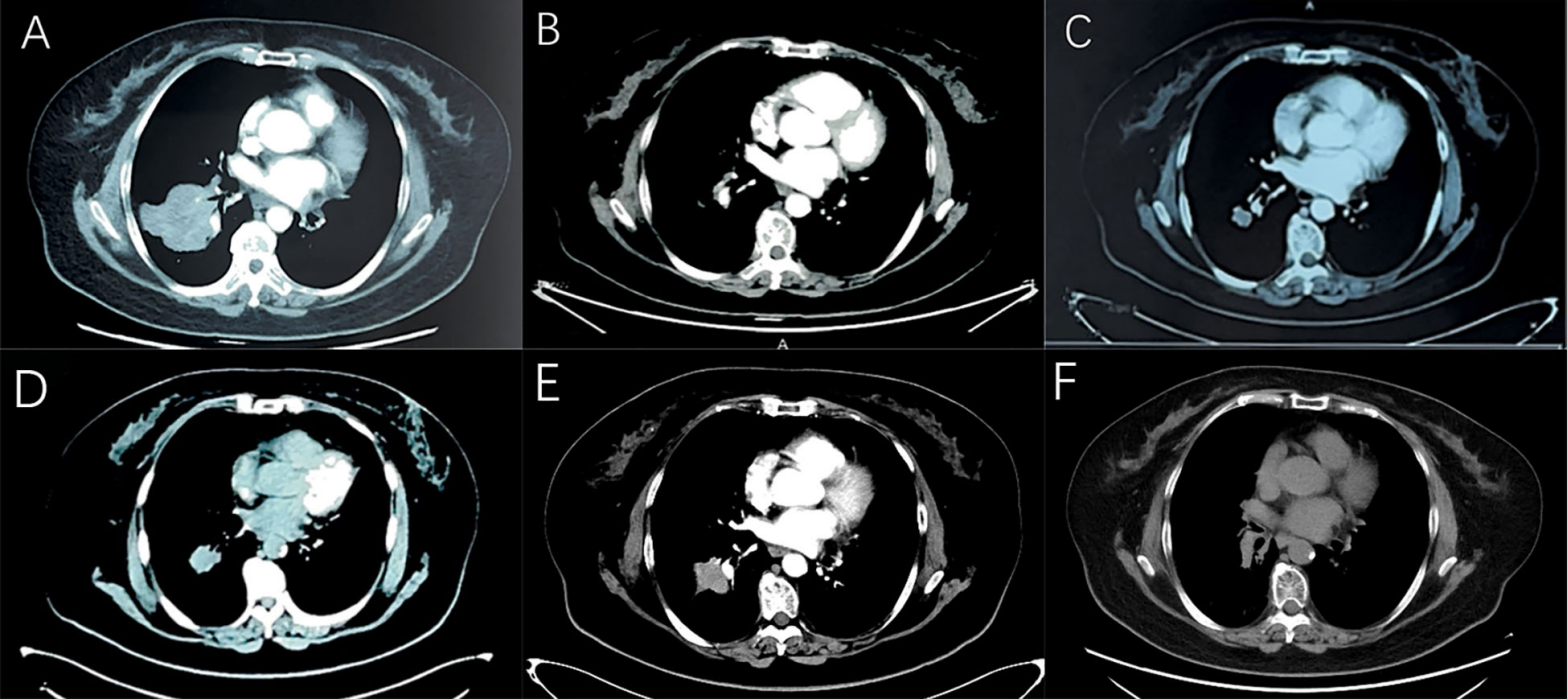
Computed tomography. **(A)** There is an obvious occupying lesion in the right lower lobe. **(B)** Targeted treatment with osimertinib resulted in a cCR. **(C)** Two years later, a space-occupying lesion reappeared, and the treatment efficacy was evaluated as PD. **(D)** Targeted treatment with furmonertinib resulted in disease progression. **(E)** Treatment with sindilizumab combined with bevacizumab and PC chemotherapy regimen resulted in disease progression. **(F)** Treatment with duvatolimab combined with EP chemotherapy regimen and local radiotherapy resulted in a PR.

**Figure 2:**
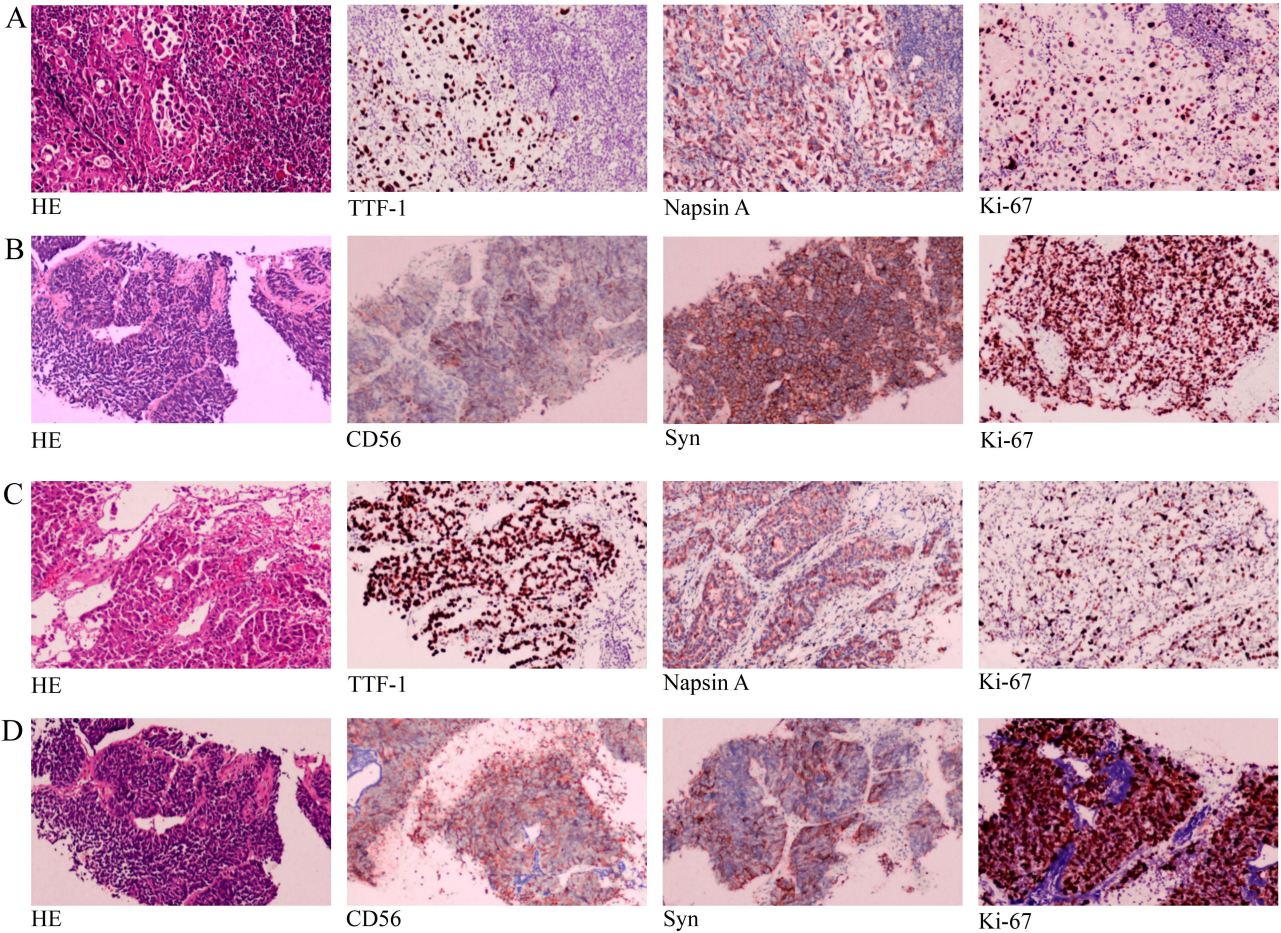
The hematoxylin and eosin (HE) and immunohistochemical (IHC) pictures: **(A)** Biopsy specimen from the right cervical lymph node revealed lung adenocarcinoma with HE staining and IHC staining for TTF-1, Napsin A and Ki-67 (20%+). **(B)** The second biopsy of the lung mass revealed SCLC with HE staining and IHC staining for CD56, Syn and Ki-67 (70%+). **(C)** Biopsy specimen from the lung mass revealed lung adenocarcinoma with HE staining and IHC staining for TTF-1, Napsin A and Ki-67 (30%+). **(D)** The second biopsy of the left supraclavicular lymph nodes revealed SCLC with HE and IHC staining for CD56, Syn and Ki-67 (80%+). All pictures were acquired with a light microscope at 100x magnification.

**Figure 3:**
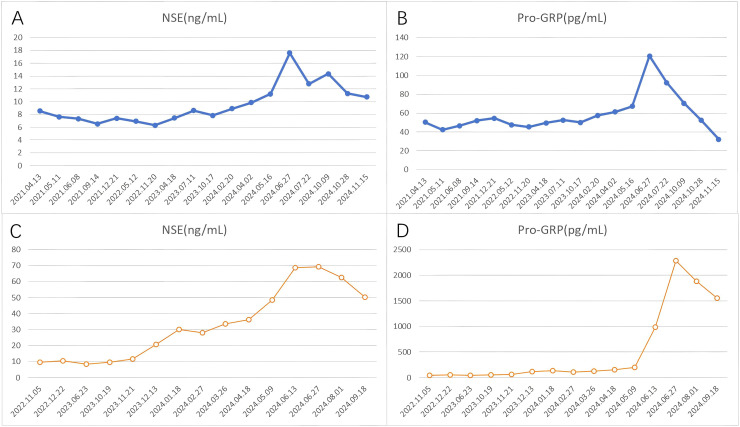
The profile of NSE and Pro-GRP in serum. **(A, B)** Case 1, **(C, D)** Case 2. NSE normal range: 0–15 ng/mL, Pro-GRP normal range: <74.4 pg/mL.

**Figure 4:**
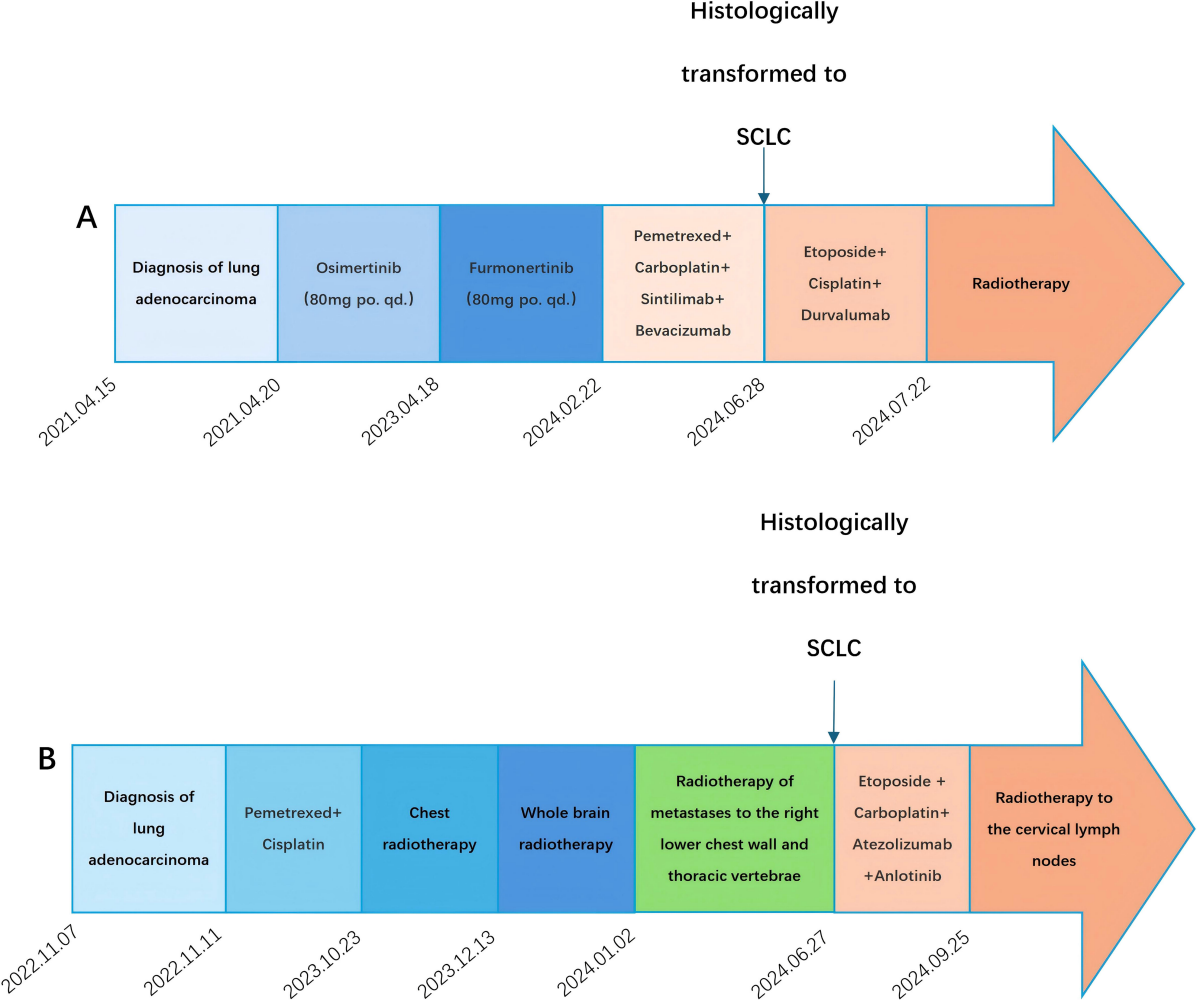
Patient‘s treatment history and medication details. **(A)** Case 1 **(B)** Case 2.

In April 2021, a 61-year-old female patient was admitted for evaluation of chest tightness and a cough. Physical examination revealed diminished breath sounds, and dry rales were heard in the right lower lung. The patient had grade 3 hypertension (very high risk), no other diseases, no family history of tumors, and no history of smoking. Chest CT revealed a mass in the lower lobe of the right lung with a distal obstructive lesion, measuring 83 mm in maximum diameter, suspected to be lung cancer (**Figure 1A**). At the same time, multiple enlarged lymph nodes were noted in the right supraclavicular fossa, mediastinum, and the right hilar region of the lung. Brain MRI revealed brain metastasis in the left occipital lobe. Tumor markers were evaluated, and all results were within the normal range: SCC 1.830 ng/mL (normal range: <2.5 ng/mL), Pro-GRP 50.40 pg/mL (normal range: <74.4 pg/mL), CEA 3.180 ng/mL (normal range: <5.5 ng/mL), CYFRA21-1 2.150 ng/mL (normal range: <3.3 ng/mL), and NSE 8.530 ng/mL (normal range: 0–15 ng/mL). An ultrasound-guided aspiration biopsy of a right cervical root lymph node was performed on April 15, 2021. Tumor tissues and their invasive tissues were collected by fine needle aspiration (FNA) and processed into formalin-fixed, paraffin-embedded (FFPE) blocks for histopathological diagnosis and molecular testing. Postoperative pathology confirmed adenocarcinoma of the lower lobe of the right lung (T2N3M1, stage IV). Immunohistochemical analysis showed: CK7 (+), CK20 (-), Villin (-), TTF-1 (+), Napsin A (+), CK5/6 (-), CR (-), CEA (+), EMA (+), and Ki-67 (approximately 20%+) (**Figure 2A**). Subsequently, genetic testing using high-throughput sequencing identified an EGFR exon 21 L858R mutation with 80% abundance, accompanied by TP53 and RB1 mutations. The patient subsequently took 80 mg of osimertinib orally daily for 2 years, with regular follow-ups during this period, achieving a clinical complete remission (cCR) (**Figure 1B**). A chest CT review in April 2023 showed an enlarged tumor with a maximum diameter of 21 mm, and efficacy was assessed as progressive disease (PD) according to RECIST criteria. (**Figure 1C**). A repeat genetic test indicated the EGFR exon 21 L858R mutation with an abundance of 23.25%. Therefore, the treatment was switched to furmonertinib 80 mg per day. A follow-up chest CT in February 2024 showed that the patient’s tumor had enlarged compared to the previous scan, with a maximum diameter of 29 mm. The efficacy assessment was recorded as PD (**Figure 1D**). Therefore, the patient was switched to PC (pemetrexed + carboplatin) chemotherapy combined with sintilimab immunotherapy and received bevacizumab for anti-angiogenesis therapy until June 2024. The June 2024 CT scan showed an increase in the size of the lung tumor compared to the February 2024 scan, with a maximum diameter of 37 mm. The efficacy assessment was recorded as PD (**Figure 1E**). Tumor markers were also assessed: Pro-GRP 120.80 pg/mL (normal range: <74.4 pg/mL), NSE 17.610 ng/mL (normal range: 0–15 ng/mL), CEA 3.020 ng/mL (normal range: <5.5 ng/mL), CYFRA21-1 3.150 ng/mL (normal range: <3.3 ng/mL), SCC 2.130 ng/mL (normal range: <2.5 ng/mL), CA 19-9 5.800 U/mL (normal range: <34 U/mL), CA 125 16.620 U/mL (normal range: <35 U/mL), and CA 15-3 24.980 U/mL (normal range: <25 U/mL). Pro-GRP and NSE were elevated, but the other markers were within the normal range (**Figures 3A, B**). To further clarify the pathological diagnosis, on June 28, 2024, we performed a CT-guided percutaneous lung aspiration biopsy on the patient. Biopsy pathology revealed infiltrative carcinoma, with immunohistochemical staining as follows: CKpan(+), TTF-1(+), Syn(+), CgA(partial+), Ki-67(approximately 70%+), CD56(+), CK5/6(-), Vimentin(-), P40(-), CK7(partial+), and NapsinA(-) (**Figure 2B**). Combined with immunohistochemical staining results, the diagnosis of small cell lung cancer transformation was confirmed in the patient. Therefore, on July 3, 2024; July 29, 2024; August 19, 2024; and October 11, 2024, the patient underwent four cycles of durvalumab immunotherapy in combination with the EP regimen (etoposide + cisplatin). After chemotherapy, the patient experienced poor tolerance, exhibiting atopic dermatitis and significant bone marrow suppression. Following aggressive symptomatic treatment, the patient’s symptoms were relieved. Volumetric-modulated arc therapy was delivered using a linear accelerator with 6-MV photons, delivering a total dose of 60 Gy in 30 fractions over six weeks, from July 22 to September 3, 2024. On follow-up CT after radiotherapy, the tumor was significantly reduced, and the efficacy evaluation showed a partial response (PR) (**Figure 1F**). After treatment, the patient’s mental status and appetite were stable, and her condition remained stable with regular follow-ups (**Figure 4A**).

### Case 2

**Figure 5:**
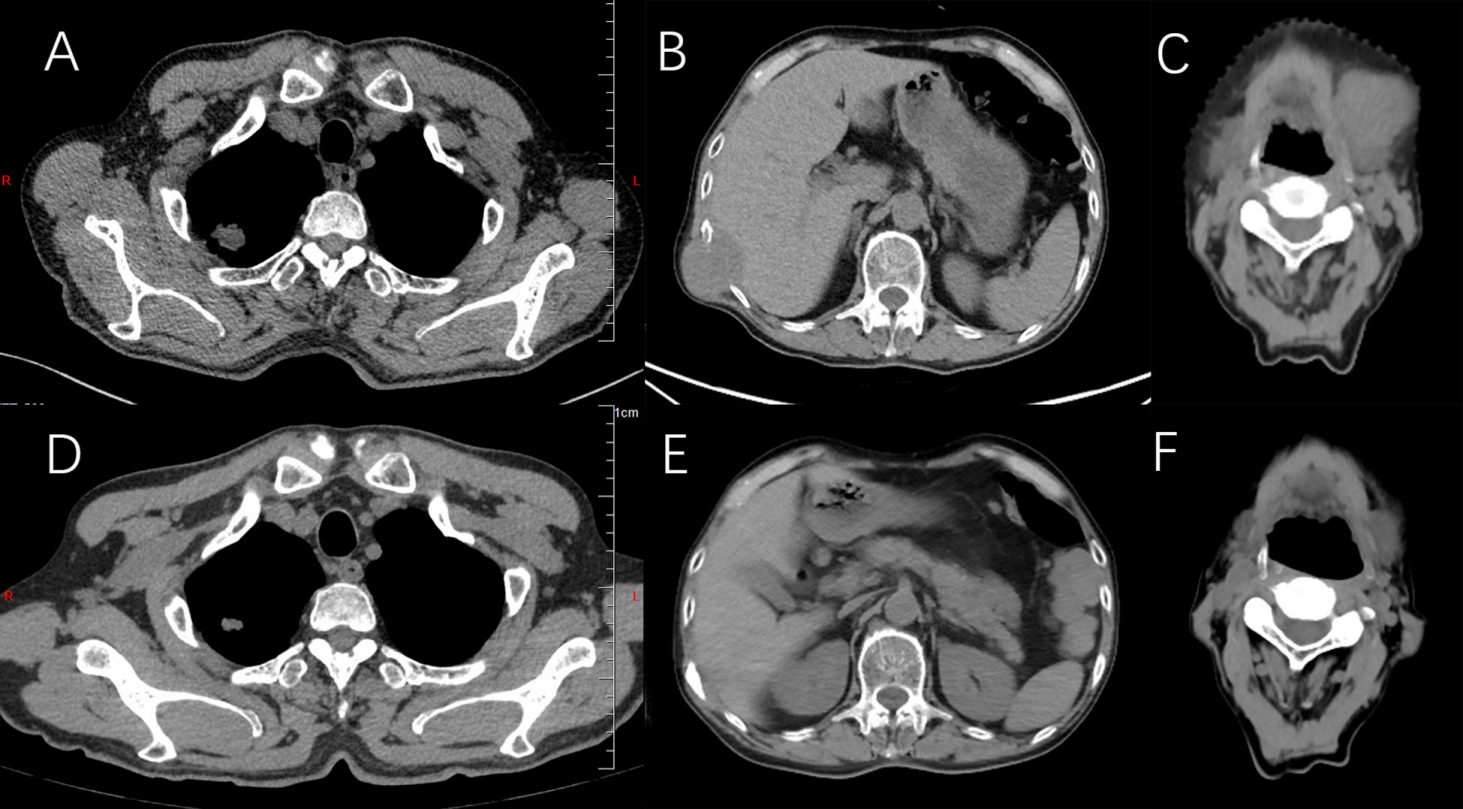
Computed tomography. **(A)** Obvious occupying lesion in the right upper lung lobe. **(B)** Metastasis occurred in the right lower chest wall and was evaluated as PD. **(C)** Metastasis occurred in the neck and was evaluated as PD. **(D)** After the PP chemotherapy regimen, the lesion decreased in size. **(E)** Radiotherapy was administered to a mass in the right lower chest wall, resulting in a significant reduction in size. **(F)** The neck mass was treated with radiotherapy, and the efficacy was evaluated as PR.

On October 3, 2022, a 64-year-old man was found to have an irregular nodular shadow, with a maximum diameter of 22 mm, in the apical segment of the right upper lobe, suspected to be a malignant tumor (**Figure 5A**). Multiple enlarged lymph nodes were also noted in the mediastinum and right hilar region on a chest examination. He was later admitted for evaluation. Physical examination revealed clear breath sounds in both lungs, with no dry or wet rales. The patient had previously undergone inguinal hernia repair, no other diseases and no family history of tumors. He had a history of heavy smoking. A CT-guided lung mass biopsy was performed on November 7, 2022. Postoperative pathology confirmed a right upper lobe adenocarcinoma (cT4N3M0, stage IIIC). Immunohistochemical analysis showed: CKpan (+), Vimentin (-), CK7 (+), CK20 (-), Villin (-), TTF-1 (+), Napsin A (+), CK5/6 (-), P40 (-), and Ki-67 (approximately 30%+) (**Figure 2C**). Later, genetic testing by high-throughput sequencing revealed mutations in PIK3CA and TP53, while EGFR, RB1, ALK, BRAF, KRAS, NRAS, MET, and ROS1 were wild-type. Due to the patient’s emaciation and poor physical condition, he was deemed unable to tolerate concurrent radiochemotherapy. As a result, the patient received 0.8 g of pemetrexed and 120 mg of cisplatin, administered over six cycles of chemotherapy. However, during the course of treatment, the patient contracted COVID-19, and chemotherapy was temporarily suspended. Chest CT on June 23, 2023, showed a reduction in size of the apical portion of the right upper lobe, with a maximum diameter of 11 mm (**Figure 5D**). The right hilar and mediastinal lymph nodes were also smaller. However, a metastatic tumor measuring 44 mm in maximum diameter was found in the right lower chest wall, involving the right 10th rib (**Figure 5B**). The efficacy assessment was recorded as PD. The patient underwent sequential chest radiotherapy for right lung cancer using a linear accelerator with 6-MV photons, delivering a total dose of 60 Gy in 30 fractions over 6 weeks, from October 23, 2023, to December 7, 2023. In November 2023, the patient experienced memory loss, dizziness, and headache and underwent evaluation for these symptoms. Cranial MRI revealed multiple intracranial metastases and whole-brain radiotherapy was initiated from December 13, 2023, to January 9, 2024, delivering a total dose of 40 Gy in 20 fractions. After treatment, the patient’s dizziness and headache resolved. A CT scan in January 2024 revealed an irregularly shaped soft tissue mass on the right side of the T11 and T12 vertebrae, with a maximum diameter of 41 mm. Metastatic tumor with bone destruction of the adjacent T11 and T12 vertebrae was suspected. The efficacy evaluation was PD. Due to severe pain and difficulty lying down caused by metastases in the right lower chest wall and thoracic vertebrae, radiotherapy was administered to these sites from January 18, 2024, to February 8, 2024, with a total dose of 54 Gy in 18 fractions. After radiotherapy, the patient’s pain was relieved, mental status improved, and appetite was good. As the patient was unable to tolerate intravenous chemotherapy due to poor physical condition, maintenance treatment with anlotinib combined with atezolizumab was administered every three weeks throughout and after the radiotherapy period. In May 2024, the patient was found to have a hard mass in the neck that gradually increased in size, with a maximum diameter of 41 mm (**Figure 5C**). Tumor control was poor. A CT scan of the abdominopelvic region was performed on June 27, 2024. The metastases in the right lower chest wall were smaller compared to previous scans, with a maximum diameter of 21 mm (**Figure 5E**). Metastases in the T11 and T12 vertebrae showed no significant changes. Multiple subcutaneous soft tissue metastases were observed in the abdominopelvic wall. Bilateral adrenal metastases were present, along with multiple soft tissue density shadows in the mesenteric area, paracolon, pararenal region, between the pancreas and stomach, retroperitoneal area, and pelvis, suggestive of metastatic tumors. Tumor progression was considered, and the efficacy evaluation was assessed as PD. Further assessment of tumor markers revealed the following results: CEA 639.800 ng/mL (normal range: <5.5 ng/mL), NSE 69.290 ng/mL (normal range: 0–15 ng/mL), Pro-GRP 2286.00 pg/mL (normal range: <74.4 pg/mL), CYFRA21-1 14.270 ng/mL (normal range: <3.3 ng/mL), SCC 2.230 ng/mL (normal range: <2.5 ng/mL), CA125 15.090 U/mL (normal range: <35 U/mL), and CA199 6.470 U/mL (normal range: <34 U/mL). CEA, NSE, Pro-GRP, CYFRA21-1, and SCC were elevated, while CA125 and CA199 remained within the normal range (**Figures 3C, D**). The patient’s condition worsened, and to further clarify the diagnosis, an ultrasound-guided biopsy of the left supraclavicular lymph nodes was performed on June 27, 2024. The pathological examination showed infiltrative carcinoma with extensive necrosis. Immunohistochemical staining revealed the following: CKpan (+), Vimentin (-), CK7 (partially +), NapsinA (-), TTF-1 (+), P40 (-), P63 (-), Syn (+), CgA (-), CD56 (+), and Ki-67 (approximately 80%+) (**Figure 2D**). Combined with immunohistochemical staining results, a diagnosis of small cell carcinoma transformation was confirmed. The patient underwent two cycles of chemotherapy with the EC regimen (etoposide + carboplatin) on July 3, 2024, and August 2, 2024. Additionally, on July 3, 2024, August 2, 2024, October 10, 2024, and November 6, 2024, the patient received four cycles of atezolizumab combined with anlotinib. From September 25, 2024, to November 5, 2024, the patient underwent radiotherapy to the bilateral cervical lymph nodes and bilateral supraclavicular lymph nodes, receiving a total dose of 60 Gy in 30 fractions. The patient developed myelosuppression following radiotherapy, which resolved after prompt symptomatic treatment. On December 28, 2024, a follow-up CT scan showed a significant reduction in the size of the neck mass, with a maximum diameter of 24 mm, indicating a PR to treatment (**Figure 5F**). The patient is currently under regular follow-up, with stable disease (**Figure 4B**).

## Discussion

Currently, two main hypotheses exist regarding the pathogenesis of SCLC transformation. Histological transformation of NSCLC to SCLC is generally considered a rare mechanism of acquired resistance to EGFR inhibitors (2). A review of the relevant literature in PubMed revealed 17 previously reported cases of sequential SCLC after adenocarcinoma, involving 7 male and 10 female patients. Ten of the patients were nonsmokers and six were smokers, with a median age of 50-60 years. All patients were treated with EGFR-TKIs and eventually underwent transformation into small cell lung cancer, with a median survival of approximately 6 to 9 months after transformation (**Table 1**). Approximately 3–10% of patients with EGFR-mutant NSCLC experience SCLC transformation following treatment with EGFR-TKIs (19). This transformation is strongly associated with inactivating mutations in the RB1 and TP53 genes, with triple-mutant adenocarcinomas (EGFR/RB1/TP53) being at higher risk of transformation to SCLC (7). The risk of transformation significantly increases to 18% in patients with an EGFR/TP53/RB1 triple mutation (20). In Case 1, genetic testing at the time of initial treatment revealed EGFR, RB1, and TP53 mutations. After more than two years of treatment with osimertinib, pathological transformation occurred. Therefore, during clinical diagnosis and treatment, the presence of triple mutations in genetic testing should raise suspicion of potential SCLC transformation in the later stages of treatment resistance. The second hypothesis suggests that SCLC transformation can occur in ALK inhibitor-treated lung cancers or in wild-type EGFR or ALK NSCLC treated with immunotherapy; however, the exact mechanism remains unclear. In Case 2, initial genetic testing showed wild-type EGFR and RB1, with only a TP53 mutation present. Despite this, the patient eventually underwent pathological transformation. The underlying mechanism remains uncertain, and further follow-up studies are needed to elucidate the process. Small cell lung cancer (SCLC) transformation is most common in EGFR-mutant NSCLC, particularly in patients with concomitant TP53 and RB1 loss of function. NSCLC with alterations in other driver genes (e.g., ALK, ROS1, RET, KRAS, etc.) may also undergo transformation, but this occurs rarely (19–21).

**Table 1:** Cases of small cell lung cancer transformed from lung adenocarcinoma.

Case	Age	Sex	Smoking history (pack years)	Initial histology/genetic mutation	TNM	Treatment	Transformed histology/ genetic mutation	Treatment after transformation	OS after transformation
Otoshi, Ryota, et al. (3)	68	M	40	ADC, EGFR 19 del, EGFR T790M mutation	NM	Osimertinib; erlotinib; osimertinib; carboplatin, paclitaxel, docetaxel, pemetrexed; S-1 monotherapy	SCLC, EGFR 19 del	EC	NA
Hui, Monalisa, et al. (4)	46	M	Smoker (specific pack-years NM)	ADC, EGFR 19 del	NM	Pemetrexed, carboplatin, zoledronate; gefitinib	SCLC, EGFR 19 del	EC	NA
Ahn, Soomin, et al. (5)	57	F	0	ADC, EGFR exon 21 L858R	T2N0M0	Operation, paclitaxel, carboplatin; iressa; afatinib	SCLC, EGFR exon 21 L858R	EP	NM
	55	F	0	ADC, EGFR 19 del	T2N3M1	Iressa	SCLC, EGFR 19 del	EP	NA
Digiacomo, Nunzio, et al. (6)	50	F	30	ADC, EGFR exon 18 mutation	NM	Osimertinib	SCLC, PDL1, EGFR exon 18 mutation, PIK3CA mutation	EC; atezolizumab	NM
Lyu, Heng-Xu, et al. (7)	65	F	0	ADC, EGFR exon 21 L858R, PTEN mutation	T2N2M1	Osimertinib, anlotinib	SCLC, EGFR exon 21 L858R, PTEN mutation	Serplulimab, EC; radiotherapy	About 6 months
Tokaca, Nadza, et al. (8)	60	F	0	ADC, EGFR 19 del	T1aN0M1a	Gefitinib; radiotherapy	SCLC, EGFR 19 del	EC; CAV; Nivolumab	About 20 months
Liu, Hao, et al. (9)	63	F	0	ADC, EGFR mutation	T2N2M1	Gefitinib	SCLC	Refuse treatment	About 1 months
Wang, Yi, et al. (10)	48	F	NM	ADC, EGFR 19 del, EGFR T790M mutation	T2aN0M0	Operation; paclitaxel, nedaplatin; gefitinib; osimertinib	SCLC, EGFR 19 del, PIK3CA mutation	EP	NM
Xue, Shuping, et al. (11)	49	F	0	ADC, EGFR 19 del	pT2N2M0	Operation; PP; Gefitinib; osimertinib	SCLC	EC	NA
Zhang, Cuicui, et al. (12)	56	M	50	ADC, EGFR 19 del, EGFR amp, RB1 and TP53 mutations	cT3N3M1a	Icotinib	SCLC, EGFR 19 del, EGFR amp, RB1 and TP53 mutations, MSH6, PMS2 amp; PD-L1(–); TMB of 15.32Muts/Mb	EC; docetaxel, icotinib, irinotecan, anlotinib, pabolizumab	About 9 months
Lin, Quan, et al. (13)	49	M	20	ADC, EGFR exon 21 L858R,	cT2aN2M1a	PP; gefitinib; cisplatin; docetaxel	SCLC	EP	About 8 months
Chowaniecova, Gabriela, et al. (14)	67	F	0	ADC, EGFR 19 del	NM	Afatinib; radiotherapy; PC; Osimertinib; docetaxel	SCLC	EC; topotecan; erlotinib	About 7 months
Zhai, Xiaoqian, et al. (15)	43	M	0	ADC, EGFR 19 del, high PD-L1 (80.9%) expression	pT2bN0M0	Operation; gefitinib; radiotherapy; pembrolizumab, pemetrexed; osimertinib	SCLC, EGFR 19 del, TP53 and RB1 mutation	EP; anlotinib; gefitinib; EC, durvalumab	About 21 months
Yang, Meng-Hang, et al. (16)	50	M	10	ADC, EGFR 19 del	T4N3M1b	Erlotinib; toripalimab, PC	SCLC, EGFR 19del and T790M (+)	EC; osimertinib	NA
Li, Xiaoxuan, et al. (17)	71	F	0	ADC, EGFR exon 21 L858R	NM	Osimertinib	SCLC, EGFR 19del, TP53 missense mutation, RB1 truncating mutation, EGFR amp, KIT amp,	Anlotinib, aumolertinib; EP, adebrelimab; osimertinib, EP, adebrelimab	NA
Liu, Yangyang, et al. (18)	38	M	0	ADC, EGFR exon 21 L858R	NM	PP, erlotinib	SCLC	EP	NM
Our case	61	F	0	ADC, EGFR exon 21 L858R, TP53, RB1	T2N3M1	Osimertinib; furmonertinib; PC, sintilimab; bevacizumab	SCLC	EP; durvalumab; radiotherapy	NA
Our case	64	M	15	ADC, PIK3CA, TP53	cT4N3M0	PP; radiotherapy; anlotinib, atezolizumab	SCLC	EC, atezolizumab, anlotinib; radiotherapy	NA

M, male; F, female; ADC, adenocarcinoma; SCLC, small cell lung cancer; NM, not mentioned; NA, not applicable; EC, etoposide plus carboplatin; EP, etoposide plus cisplatin; PP, pemetrexed plus cisplatin; amp, amplification; PC, pemetrexed plus carboplatin; OS, overall survival; TMB, tumor mutational burden; CAV, cyclophosphamide, doxorubicin plus vincristine.

Pathological transformation should be considered in NSCLC patients who show insensitivity to treatment during clinical practice. Additionally, a rapid increase in peripheral serum NSE and Pro-GRP levels has been identified as a predictive marker for pathological transformation to SCLC (18, 22). In the two cases we reported, disease progression was observed during treatment, accompanied by a rising trend in NSE and Pro-GRP levels in the patient’s serum. Subsequent biopsies confirmed pathological transformation in both cases. Therefore, dynamic monitoring of NSE and Pro-GRP levels during treatment, combined with timely biopsy when necessary, is crucial for improving diagnosis, treatment, and patient prognosis.

T-SCLC typically exhibits rapid disease progression and poor prognosis due to its aggressiveness and the limited availability of effective therapeutic options, making the search for new treatments essential. T-SCLC shares similar pathological morphology, molecular features, clinical presentations, and drug sensitivity with SCLC, suggesting that they may benefit from similar therapeutic strategies (23). One potential mechanism underlying the histological transition from EGFR-mutant NSCLC to SCLC is lineage plasticity, which enables the dedifferentiation of cancer cells from an epithelial to a neuroendocrine lineage in response to EGFR-TKI treatment (24). Neuroendocrine differentiation is also observed after pathological transformation of NSCLC, and this phenotype tends to be sensitive to chemotherapy (18). Platinum-based chemotherapy (cisplatin or carboplatin) combined with etoposide remains the primary treatment regimen for most patients diagnosed with T-SCLC (28). A retrospective study used platinum-etoposide to treat transformed SCLC, in which the clinical response rate among 46 patients reached 54% at transformation, with the median progression-free survival of only 3.4 months (range: from 2.4 to 5.4 months) (19). In addition to EC regimen chemotherapy, treatment options for T-SCLC include paclitaxel-based chemotherapy, anti-angiogenic therapy with anlotinib, and local radiotherapy (25, 26). A real-world study included 29 patients who developed SCLC transformation following EGFR-targeted therapy. The analysis indicated that compared to chemotherapy alone, the combination of chemotherapy and targeted therapy improved objective response rates and PFS, although it did not significantly extend OS. Anti-angiogenic therapy and local radiotherapy can prolong OS after transformation (26). A previous study reported 18 patients who received anti-angiogenic agents in the first- or second-line after SCLC transformation with anlotinib being most frequently administered. The study demonstrated that anlotinib can treat transformed SCLC patients and achieve longer OS than those who did not receive anti-angiogenic agents (26). Another study validated this result using EGFR mutant transformed SCLC patients and achieved an ORR of 66.7% and a median PFS of 6.2 months (27). Tomic, Kresimir, et al., reported that the median time to T-SCLC transformation in EGFR-mutant NSCLC patients treated with TKIs is approximately 17 months, with a median overall survival of 10 months (28). For patients whose NSCLC transformed to SCLC following immunotherapy, overall survival times varied widely, ranging from 2 to 16 months (29). In case 1, a pathological transformation was observed during re-examination after more than 2 years of treatment with osimertinib. However, we considered that the patient’s pathological transformation might have occurred earlier. Therefore, re-biopsy is necessary in cases of EGFR-TKI resistance. Zhang, Chan-Yuan, et al. retrospectively analyzed the efficacy of immunotherapy combined with chemotherapy in T-SCLC. The results indicated that this approach could be a therapeutic option for patients with T-SCLC and might benefit from the addition of anti-angiogenic therapy (30). Case 1 was treated with 4 cycles of immunotherapy and anti-angiogenic therapy combined with EP regimen chemotherapy after the diagnosis of T-SCLC. Due to the limited lung lesions in case 1, local tumor radiotherapy was administered to control the lesions. Follow-up CT showed that the lesions remained controlled, and the patient’s general condition was still good, suggesting the treatment was effective. Case 2 was treated with the EC regimen chemotherapy for 2 cycles after the diagnosis of T-SCLC, and the tumor regressed significantly. Both of our patients were treated aggressively, and tumor control was achieved. Therefore, we believe that the treatment of T-SCLC should include chemotherapy based on the EC regimen, combined with immune and anti-angiogenic therapy, and radiotherapy should be administered to local metastatic foci. If the patient’s physical condition permits, it is recommended to actively administer comprehensive treatment to improve the quality of life and the patient’s prognosis.

## Conclusion

This study presents two cases of lung adenocarcinoma in which patients experienced disease progression after different treatments, both of which were subsequently pathologically confirmed to have transformed into SCLC. These findings emphasize the importance of dynamic tumor marker monitoring and re-biopsy in cases of NSCLC treatment resistance. Compared to typical SCLC, T-SCLC progresses more rapidly. Upon clinical confirmation of SCLC transformation, early development of an individualized treatment plan, along with regular follow-ups to promptly adjust therapeutic strategies, is crucial. The limitations of current diagnostic and therapeutic approaches highlight the urgent need for improved T-SCLC guidelines and clinical practices. A multidisciplinary approach is vital to better understand the disease and tailor effective treatment strategies.

## Data Availability

The original contributions presented in the study are included in the article/supplementary material. Further inquiries can be directed to the corresponding author.
